# Building bridges, not walls: spinal cord regeneration in zebrafish

**DOI:** 10.1242/dmm.044131

**Published:** 2020-05-27

**Authors:** Valentina Cigliola, Clayton J. Becker, Kenneth D. Poss

**Affiliations:** 1 Department of Cell Biology, Duke University Medical Center, Durham, NC 27710, USA; 2 Regeneration Next, Duke University, Durham, NC 27710, USA

**Keywords:** Regeneration, Spinal cord, Zebrafish

## Abstract

Spinal cord injury is a devastating condition in which massive cell death and disruption of neural circuitry lead to long-term chronic functional impairment and paralysis. In mammals, spinal cord tissue has minimal capacity to regenerate after injury. In stark contrast, the regeneration of a completely transected spinal cord and accompanying reversal of paralysis in adult zebrafish is arguably one of the most spectacular biological phenomena in nature. Here, we review reports from the last decade that dissect the mechanisms of spinal cord regeneration in zebrafish. We highlight recent progress as well as areas requiring emphasis in a line of study that has great potential to uncover strategies for human spinal cord repair.

## Abstract (precis)

**Summary:** Unlike mammals, teleost fish are capable of efficient, spontaneous recovery after a paralyzing spinal cord injury. Here, we highlight the major events through which laboratory model zebrafish regenerate spinal cord tissue.

## Introduction

Movement is a fundamental method to interact with the world around us. Under normal conditions, our central nervous system (CNS) sends signals through descending neural tracts to control movement. This process occurs automatically such that it is difficult to conceive what life would be like if those tracts were interrupted.

**Fig. 1.**
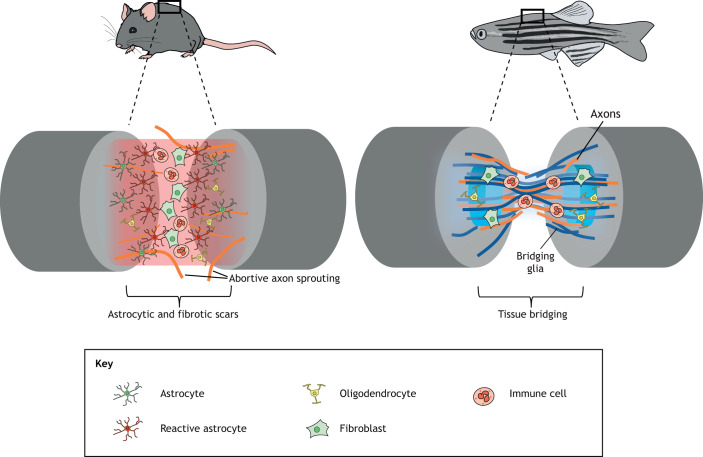
**Different responses to spinal cord injury (SCI) in zebrafish and mammals.** Representation of different cellular events occurring after SCI in zebrafish and mice. Upon SCI in mammals, a complex cascade of events occurs, leading to the formation of a scar at the lesion site constructed by stromal-derived fibroblasts, inflammatory immune cells and hypertrophic astrocytes. The scar impedes the regrowth of spared axons. Conversely, in zebrafish, injury induces the infiltration of immune cells followed by bridging of glial cells and axonal tracts, leading to functional regeneration.

Spinal cord injury (SCI) in mammals causes massive cell death. Severed distal axons that have lost contact with neuronal cell bodies dissolve through a stereotyped process known as Wallerian degeneration. Occasionally, proximal axonal tracts survive and sprout, but, in the vast majority of cases, they fail to regenerate or re-innervate appropriate targets (Cajal, 1928). A major physical barrier to axon regrowth is the formation of a heterogeneous mass of tissue mainly consisting of reactive astrocytes, fibroblasts and inflammatory immune cells, commonly referred to as a glial scar (Fig. 1). To complicate matters, local astrocyte loss can alter neuronal ion homeostasis, and oligodendrocyte deficiency contributes to poor myelination and impaired axonal activity (Grossman et al., 2001; Thuret et al., 2006; Bradbury and Burnside, 2019; Courtine and Sofroniew, 2019).

Because of this poor capacity for the CNS to regenerate disrupted circuits, and because there are no effective strategies to boost this capacity, most affected individuals remain paralyzed for their entire lives. SCI, and all complications associated with impaired sensory and motor function, currently affect approximately 291,000 people in the United States, with an average age at injury of about 43 years (National Spinal Cord Injury Statistical Center, 2019). For those paralyzed individuals, the discovery of methods to re-establish functional neuronal connections is critical, as these could be coupled with electrostimulatory and engineering approaches to therapeutically relieve paralysis.

**Fig. 2.**
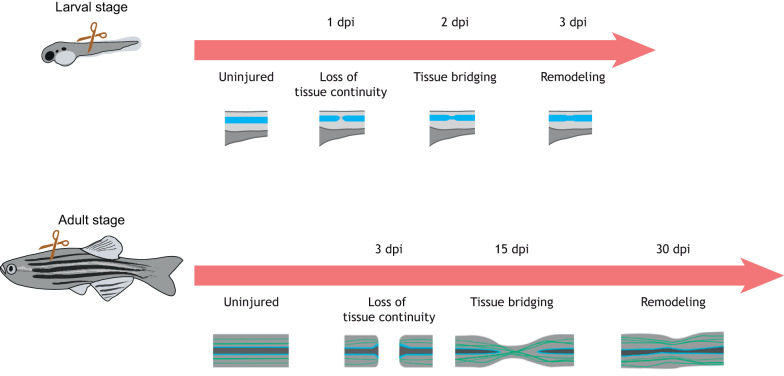
**Time course of spinal cord regeneration in zebrafish.** (Top) In zebrafish larvae, spinal cord transection destroys axonal and glial connections, eliciting the formation of a tissue bridge that spans the injury epicenter by 2 days post-injury (dpi). A remodeling phase follows at 3 dpi. (Bottom) A similar response to injury is observed in adult zebrafish. By 15 dpi, some axonal and glial processes have traversed the injury site. Subsequent remodeling reconstitutes structure.

Unlike humans or commonly studied mammalian model systems, a number of vertebrates can regenerate crushed or transected spinal cord tissue at the adult stage. Regrowth of severed axons, repair of neuronal circuits and functional recovery to full movement capacity have been observed in tadpole-stage frogs (Edwards-Faret et al., 2017), in adult salamanders (Butler and Ward, 1967; Piatt and Piatt, 1958), to some extent in certain reptiles (Rehermann et al., 2009; Simpson, 1964), in lampreys (Cohen et al., 1986; McClellan, 1990; Oliphint et al., 2010; Rovainen, 1976; Selzer, 1978) and in teleost fish species (Becker et al., 1997; Bernstein, 1964). Researchers employing amphibians and reptiles are developing, or are positioned to develop, numerous transgenic and genetic tools for dissecting the mechanisms of these events (Tazaki et al., 2017; Jacyniak et al., 2017). In this article, we focus on studies in zebrafish, which can be experimentally manipulated by arguably the most mature toolset among vertebrates with elevated regenerative capacity. Zebrafish have other advantages, such as their relatively short generation times compared to other non-mammalian species that can be genetically modified (e.g. salamanders) and low maintenance costs (Becker et al., 1997; Hui et al., 2010) (Fig. 2).

Spinal cord regeneration in zebrafish is complex, involving inflammation, cell death, cell migration, cell proliferation, neurogenesis, axonogenesis, and tissue- and circuit-level remodeling (Hui et al., 2014). Many questions inherent to the processes that occur during spinal cord regeneration are still unresolved and, perhaps surprisingly, are the subject of only a handful of research groups worldwide. Here, we present an overview of the past decade of experiments assessing mechanisms of spinal cord regeneration in larval and adult zebrafish, keeping in mind the recognized potential pitfalls of morpholino-based studies. We focus on the molecular signals implicated in hallmark events through which lost tissue is recovered. We anticipate that these discoveries will direct future investigations and suggest avenues to boost regeneration in mammalian species.

## Establishing a progenitor pool for new neurons

Studies over the past decade, reviewed below, have indicated that, upon injury, the adult zebrafish spinal cord triggers proliferation of the resident neural progenitors and their subsequent differentiation into new neurons that are able to integrate into the existing circuitry (Ghosh and Hui, 2016). Interestingly, neurons are produced in excess upon injury: this is true for serotonergic neurons, the numbers of which increase fivefold compared to those in uninjured control animals (Kuscha et al., 2012b), and motor neurons, which are generated in excess compared to what is later stabilized in the spinal cord (Reimer et al., 2008). These phenomena indicate that the level of neurogenesis upon injury is not tightly regulated.

Neurons have been proposed to arise from a special type of cell with its soma lining the spinal cord's central canal and long radial processes contacting the pial surface with a foot-like structure. These cells, first identified in the brain, share some features with radial glia, such as expression of the astrocyte marker glial fibrillary acidic protein (Gfap) and of aquaporin-4 and glutamine synthase. They possess functions such as sealing the blood-brain barrier or regulating ion homeostasis, while also lining the central canal and being ciliated (Becker and Becker, 2015). Because of this hybrid nature and function, these progenitors are referred to as ependymo-radial glial cells (ERGs). The idea that ERGs would be the major source of new neurons in the CNS upon injury was first proposed after observing that they normally proliferate slowly, but respond to injury with a strong increase in proliferation in the adult zebrafish brain (Grandel et al., 2006; Rothenaigner et al., 2011). Using a cell-labeling strategy in adult newts, Berg et al. identified ERGs as a source of new tyrosine hydroxylase-positive (TH^+^) neurons after ablation of dopaminergic cells by stereotaxic injection of 6-hydroxydopamine (Berg et al., 2010). The possibility that ERGs could behave in a similar manner in adult zebrafish was first proposed in 2008, when Reimer et al. followed labeled *olig2*-expressing ERGs in a Tg(*olig2*:EGFP) transgenic zebrafish line and observed that, after SCI, some of the newly generated neurons identified by HB9 (also known as Mnx1) expression (a marker of motor neurons) also expressed EGFP (Reimer et al., 2008). More direct evidence that ERGs give rise to CNS neurons came in 2011, when Kroehne et al. genetically marked adult zebrafish brain ERGs for the first time using a Cre-LoxP system to irreversibly trace their progeny. In this study, the authors used a transgenic line expressing a bicistronic mRNA coding for mCherry and CreER^T2^ recombinase under the control of the zebrafish *her4.1* promoter, with expression increasing in proliferating ERGs upon injury. These fish were crossed to a line allowing irreversible, Cre-released EGFP expression upon tamoxifen administration. After a stabbing brain lesion, the newly generated neurons were EGFP labeled, indicating that they were derived from ERGs (Kroehne et al., 2011).

Of note, a non-radial glial cell population with stem cell properties named ‘boundary cells’ or ‘progenitor pools’ has been reported to give rise to ERGs and neurons in the zebrafish telencephalon (de Oliveira-Carlos et al., 2013). It is important to determine which progenitor population(s) exist in spinal cord and how they behave upon injury, even if their contribution to regeneration is expected to be minor. ERGs expressing the transcription factor *foxj1a* and proliferating in response to injury have been identified in zebrafish larvae and adults, with their expansion proposed to depend on Hedgehog (Hh) signaling. Indeed, treatment of zebrafish larvae with the Hh inhibitor cyclopamine after spinal cord transection reduced *foxj1a* transcript levels and decreased ERG proliferation (Ribeiro et al., 2017). Notably, ERGs also display regional differences, i.e. they have different transcription factor expression profiles according to their dorsoventral position in the central canal (Becker and Becker, 2015), an indicator of the specific neuronal subtype they will give rise to, as discussed below.

### Motor neuron regeneration

Motor neurons control muscle movements by transmitting impulses directly from the spinal cord to skeletal muscle. As reviewed here, their regeneration is influenced by an array of local and remote signals.

#### Transcriptional regulators

Lineage-tracing experiments have revealed that a subset of ERGs lining the central canal in dorsoventral positions increase expression of *olig2*, *nkx6*.1 and *pax6* (of which zebrafish has two orthologs, *pax6a* and *pax6b*) upon SCI, where they proliferate and act as motor neuron progenitors in adult zebrafish (Reimer et al., 2008, 2009). This same expression signature has also been observed in zebrafish larvae upon nitroreductase technology-enabled ablation of motor neurons, suggesting that their targeted loss is sufficient to trigger regeneration from ERGs (Ohnmacht et al., 2016).

Consistent with cells in the ependyma that possess neural stem cell properties, Guo et al. found that mRNA coding for the transcription factor gene *sox11b* is localized in a subset of cells lining the central canal upon adult spinal cord transection, as well as in newly differentiated neurons. Sox11b could ostensibly act by upregulating the expression of the pro-neural basic helix-loop-helix transcription factor *ascl1a* and the neural stem cell-associated gene *nestin*, both of which participate in neuronal differentiation during embryogenesis (Guo et al., 2011). Aside from Sox11b, Ogoi et al. reported the upregulation of Sox2 in ependymal cells following spinal cord transection in adult zebrafish and suggested a role for this transcription factor in proliferation. Six to 9% of Sox2-expressing cells also expressed the neuronal marker HuC/D (also known as Elavl3/4), indicating that Sox2-positive cells are contributing to regenerative neurogenesis (Ogai et al., 2014). It will be important to further elucidate the roles of Sox11b and Sox2, and of other transcriptional complexes and their corresponding target genes that constitute the neurogenic programs activated by injury.

#### Signaling pathways

As is often the case in the context of regeneration, the signals first deployed in developing embryos are re-engaged during spinal cord regeneration, with a handful implicated in motor neuron regeneration. Among these, Hh signaling appears to have a crucial role, as transcript levels for the receptor *patched 1* and the co-receptor *smoothened* are increased in **Olig2^+^*/*Nkx6.1^+^*/*Pax6^+^** progenitors upon injury. Blockade of Hh signaling with cyclopamine impairs motor neuron regeneration in adult zebrafish (Reimer et al., 2009). As might be expected, zebrafish have also been studied to elucidate the molecular influences that restrict, rather than promote, regeneration. For example, Notch signaling, as assessed by *in situ* hybridization of Hairy-related (Her) genes, is reactivated upon injury in adults, predominantly in Olig*^+^* progenitor cells that give rise to HB9-expressing motor neurons. Induced transgenic expression of an activated Notch1a receptor reduces motor neuron regeneration, concomitant with attenuated neural progenitor proliferation. In this same study, blockade of Notch signaling with the gamma-secretase inhibitor (2S)-N-[(3,5-difluorophenyl)acetyl]-L-alanyl-2-phenyl]glycine 1,1-dimethylethyl ester (DAPT) conversely increased motor neuron generation (Dias et al., 2012).

In addition to Notch and Hh, fibroblast growth factor (Fgf) signaling has recently been implicated in adult motor neuron regeneration: Fgf3 has been described to direct neurogenesis of *islet1* (also known as *isl1*)-expressing motor neurons and to induce axonogenesis in cMet (also known as Met)-expressing motor neurons. The effects of Fgfs are likely to be mediated through the Mapk pathway and appear to be conserved in mammalian cells (Goldshmit et al., 2018). In addition to these pathways, Briona et al. used an inducible fate-mapping system to show that Gfap^+^ cells in zebrafish larvae display neurogenic potential upon injury that depends upon the levels of Wnt/β-catenin signaling (Briona et al., 2015).

#### Inflammatory signals

The immune system is now well recognized as a key regulator of tissue regeneration, acting either as a pro- or an anti- regenerative influence (Eming et al., 2017; Karin and Clevers, 2016). With respect to zebrafish spinal cord regeneration, suppressing the inflammatory response that follows SCI in larvae with the immunosuppressant dexamethasone reduces motor neuron regeneration (Ohnmacht et al., 2016). In adult zebrafish, elegant genetic studies have dissected the roles of regulatory T cells (Tregs) in spinal cord regeneration. Tregs are characterized by expression of *foxp3*, and their genetic ablation disrupts normal regeneration of spinal cord tissue in zebrafish. Tregs are proposed to act by inducing proliferation of Sox2*^+^* neural progenitors, and they appear to remain near or in direct contact with HuC/D-expressing newly formed neurons after injury. One of the likely roles of Tregs is the production of the neurogenic factor Neurotrophin 3, as systemic delivery of this factor partially rescues the regeneration defects observed in fish lacking Tregs (Hui et al., 2017; Ogai et al., 2014).

#### Neurotransmitters

Dopamine (DA) has been reported to act as a remote signal affecting motor neuron regeneration after SCI in adult zebrafish. DA is proposed to be released by brain-derived dopaminergic TH1^+^ axons sprouting after spinal cord transection in areas rostral to the lesion and in close proximity to Olig2*^+^* ERGs. Reimer and colleagues reported that endogenous DA is required for the regeneration of motor neurons, and that intraperitoneal injections of a DA agonist increase the proportion of regenerated motor neurons. DA action appears to be mediated through the D4 receptor, a negative regulator of the cyclic adenosine monophosphate (cAMP)/protein kinase A (PKA) signaling pathway. Supporting this hypothesis, inhibition of cAMP in larvae using the SQ22536 inhibitor significantly increased the development of motor neurons. Conversely, increasing cAMP levels through rolipram administration reduced motor neuron numbers. As cAMP controls PKA activity, which negatively regulates Hh signaling, the authors suggested that DA likely influences spinal cord neurogenesis by feeding into Hh signaling (Reimer et al., 2013). Similar to dopamine, serotonin, a neurotransmitter supplied to the spinal cord mainly by descending axons from the brain, induces regeneration of motor neurons from ERGs, which express serotonin receptors. Ablation of serotonergic axons in adult zebrafish through toxins abolished motor neuron regeneration, and, conversely, increasing serotonin levels through intraperitoneal injections increased the number of regenerated motor neurons (Barreiro-Iglesias et al., 2015). Although there is more to elucidate regarding the direct and/or indirect effects of DA and serotonin on ERGs, these studies suggest an intriguing crosstalk between brain-derived neuronal projections and ERGs.

### Interneuron regeneration

Bilateral movement is coordinated by interneurons. V0-V2 classes of interneurons have been directly identified in zebrafish, whereas the presence of V3 interneurons is still uncertain. Interneuron regeneration upon SCI has been evaluated using transgenic zebrafish in which the regulatory sequences upstream of *vsx1*, an early marker for undifferentiated V2 interneurons, direct EGFP expression (Batista et al., 2008; Kuscha et al., 2012b). While expression of this transgene is not detected in the uninjured spinal cord, large numbers of EGFP-expressing V2 cells are evident in the mediolateral ependymal region upon injury. These interneuron progenitors enter the cell cycle and have been proposed to be newly generated from a specific ERG population co-expressing the markers Pax6 and Nkx6.1, and are located more dorsally in the ependymal region with respect to the progenitors giving rise to motor neurons (Kuscha et al., 2012b). While these studies are interesting, permanent cell lineage-tracing tools allowing conditional or inducible tagging of specific cell types to track their fate changes over time are needed to demonstrate the full differentiation sequence of ERGs into interneurons. In a separate study, Briona and Dorsky reported the presence of a cell population in the ependymal region of the spinal cord that expresses the transcription factor *dbx1a*. Interestingly, cells expressing *dbx1a* (one of the two zebrafish *dbx1* orthologs) exhibit a proliferative and neurogenic response to injury in zebrafish larvae, as they incorporate 5′-bromo-2′-deoxyuridine (BrdU) and express the neuronal marker HuC/D (Briona and Dorsky, 2014). The neuronal subtype derived from *dbx1a*^+^ cells remains unknown, as genetic fate-mapping studies have not been performed to trace these cells. However, studies performed in mice found that *Dbx1*-expressing cells give rise to V0 and V1 interneurons during development (Pierani et al., 2001). Consequently, one can speculate that *dbx1*^+^ cell-derived neurons belong to this subpopulation. In addition, whether cells expressing *dbx1* act as progenitors upon injury in adult zebrafish is unknown.

### Serotonergic and dopaminergic neuron regeneration

Dopaminergic and serotonergic systems play an important role in modulating spinal locomotor circuits. Upon spinal cord transection in adult zebrafish, TH1^+^ (mainly dopaminergic) and serotonin receptor (5-HT^+^)-positive (serotonergic) terminals and cells undergo major changes. TH1^+^ cell bodies are generally located in the brain and project into the spinal cord. Spinal cord lesions induce changes in TH1^+^ terminal varicosities that are regenerated, although without re-establishing the same complexity as before injury. Conversely, 5-HT^+^ varicosity indices are 80% higher than in unlesioned animals at 6 weeks post-injury. In addition, unlike TH1^+^ cell bodies, 5-HT^+^ cell bodies are newly generated after injury at the lesion site (Kuscha et al., 2012a). 5-HT^+^ cells originate from ERGs located around the central canal, in a region comprising the Olig2 ^+^ zone and a more ventral zone expressing Nkx6.1 and Sonic hedgehog a (Shha), as assessed by 5-HT antibody staining in *olig2*:GFP or *shha*:GFP reporter lines (Kuscha et al., 2012a,b; Reimer et al., 2009).

### Regeneration of undefined neuronal subtypes

**Fig. 3.**
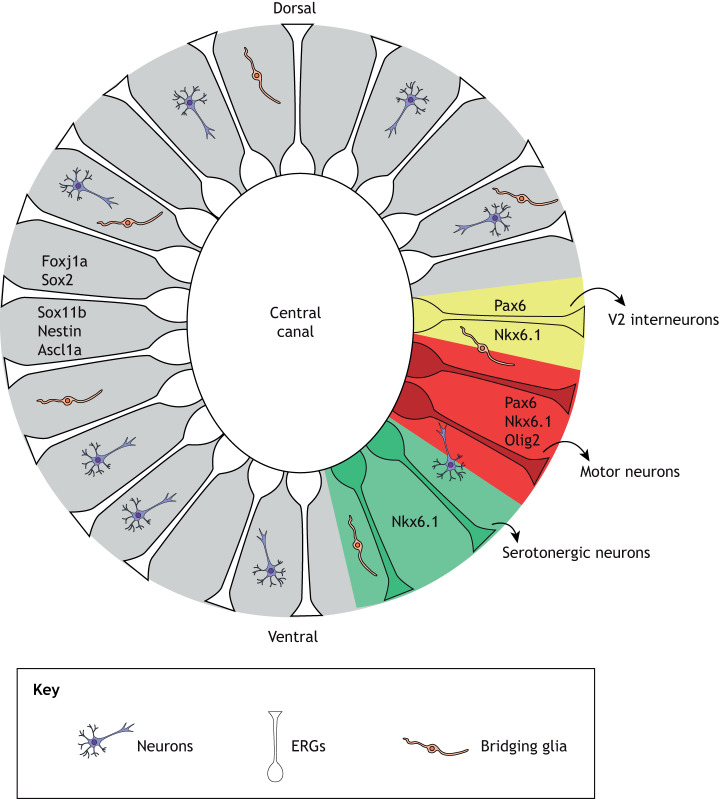
**ERG progenitor subpopulations.** A cross section of the spinal cord, illustrating the proposed ERGs organized in compartments that give rise to different neuron types. Transcription factors in the gray zones are common to all ERGs. Pax6- and Nkx6.1-expressing ERGs (yellow zones) give rise to V2 interneurons, ERGs expressing Pax6, Nkx6.1 and Olig2 (red zones) give rise to motor neurons, and ERGs only expressing Nkx6.1 (green zones) give rise to serotonergic neurons.

**Fig. 4.**
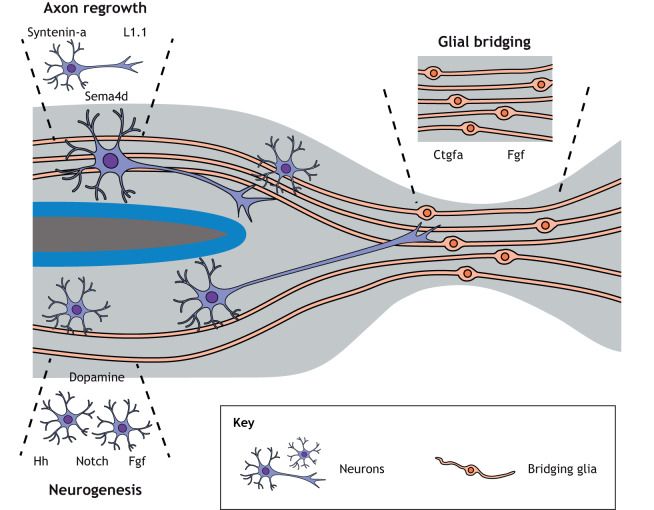
**Signals inducing neurogenesis, glial bridging and axon regrowth after SCI.** Several signaling factors have been implicated in the regrowth of axons (Syntenin-a, L1.1, Sema4d), neurogenesis (dopamine, Notch, Hh, Fgf), and formation of a glial bridge (Ctgf, Fgf) after SCI in zebrafish. For some of these (i.e. dopamine, Notch, Fgf), unexpected similarities can be observed among vertebrates with different capacities for spinal cord regeneration.

Several additional molecular cues have been shown to affect neurogenesis upon spinal cord transection, although for some of them the neuronal subtype they give rise to remains undefined. Nelson et al. reported that glucocorticoid (GC) signaling negatively affects neurogenesis by acting on ependymal glia. They found that expression of the GC receptor Nr3c1 diminishes upon spinal cord transection in ependymal glia that surround the central canal in larval zebrafish, as assessed by immunohistochemistry, and that increasing GC signaling levels with agonists decreases the proportion of proliferating HuC/D-expressing cells. Interestingly, Nr3c1 expression follows the opposite direction in rat ependymal glia that surround the central canal, where its levels increase upon injury, determined again by immunohistochemistry. This observation suggests a difference between zebrafish and mammals that might be of interest (Nelson et al., 2019). Taken together, it appears that, as described in many studies of spinal cord development (Cardozo et al., 2017), the combination of transcription factors expressed in different dorsoventral progenitor domains of the spinal cord defines the subtype of daughter neuron that regenerates after injury (Figs 3 and 4).

## The glial cell paradigm

A well-described barrier to mammalian spinal cord regeneration is astrocytic gliosis at the lesion site that leads to formation of a glial scar, which physically impedes axonal regrowth (Hu et al., 2010; Silver and Miller, 2004; Stichel and Müller, 1998). Glial cell responses to SCI in zebrafish are thought to fundamentally differ from those in mammals. Goldsmith and colleagues proposed a model in which SCI triggers proliferation of nearby glial cells and their migration to the lesion site, followed by their elongation along the anteroposterior axis and acquisition of a bipolar morphology (bridging glia). At 2-3 weeks post-injury, glial cells elongate further, forming a bridge between the two transected spinal cord ends that could serve as scaffold for axons to regrow across the lesion. Dismantling and remodeling of the glial bridge would restore the central canal and re-establish spinal cord morphology (Goldshmit et al., 2012).

Glial bridge formation depends on signaling through Fgf receptors, which affects the proliferation, migration and onset of differentiation of glial cells. Disruption of Fgf signaling post-SCI in adult zebrafish by induced expression of a dominant-negative Fgf receptor, or by treatment with the pharmacological inhibitor SU5402, blocked axonal regeneration 3 weeks post-SCI. Conversely, zebrafish with mutations in *spry4*, a known cell-autonomous inhibitor of Fgf signaling, or those injected intraperitoneally with FGF8, demonstrated accelerated glial bridging across the lesions and accumulation of regenerating axons (Goldshmit et al., 2012).

More recently, Mokalled et al. reported the involvement of connective tissue growth factor (*ctgf*, or *ccn2*), a component of the extracellular matrix (ECM) with no known extracellular receptor, but with a role in multiple signaling pathways, in glial bridge formation in adult zebrafish. One of the two zebrafish *ctgf* orthologs, *ctgfa* (also known as *ccn2a*), is induced at the spinal cord lesion site in a limited set of early bridging glia, designated ‘pioneer glia’, as well as in other tissues. A viable *ctgfa* mutant strain showed defects in glial bridging and spinal cord regeneration, whereas induced transgenic overexpression of the full-length *ctgfa* or its C-terminal domain alone increased glial bridging and axon growth. Interestingly, a one-time application of human recombinant CTGF protein at the lesion improved spinal cord regeneration in *ctgfa­* mutant zebrafish (Mokalled et al., 2016). The key next steps in this line of research include defining additional features of pioneer glia, and determining how Ctgf might be polarizing glia and instigating bridge morphogenesis. Of note, cultured primate glial cells also elongate and acquire a bipolar morphology when stimulated with exogenous human FGF2, confirming the conserved role of Fgf signaling (Goldshmit et al., 2012). Whether Fgf and Ctgf could be used as possible pro-regenerative factors in mammals remains to be fully explored, noting that their systemic delivery is likely to have limited or undesirable effects compared with more targeted delivery approaches. Wang et al. reported that small interfering RNA (siRNA) knockdown of Ctgf in the injured rat spinal cord reduced glial scar formation post-injury (Wang et al., 2018). The cellular basis of this improvement, which contrasts the *ctgfa* loss-of-function results in zebrafish, is unclear, and – to our knowledge – the effects of excess Ctgf application on mammalian SCI is unknown.

The regenerating axons bridge the lesion site in a manner that spatially and temporally correlates with the presence of bridging glia, yet the idea that the lesion site is traversed only after a glial bridge is established is controversial. Indeed, nitroreductase-enabled ablation of Gfap*^+^* glial cells did not affect axonal regrowth in zebrafish larvae (Wehner et al., 2017), meaning that regeneration could occur in the absence of (Wehner et al., 2017) or before (Dervan and Roberts, 2003) the formation of a glial bridge. This was supported by simultaneous time-lapse video microscopy imaging of axonal and glial processes, leading to the observation that most axons enter and cross the lesion site independently of glial processes (Briona et al., 2015). Along this line, work in goldfish led to the hypothesis that glial cells trail behind new axons, rather than leading their regeneration (Nona and Stafford, 1995). Although the glial ablation experiment provides good evidence that axons grow independently from the glial bridge across the lesion site, one cannot rule out the possibility that a few glial cells that escaped ablation are sufficient to form a bridge (Fig. 4). It would be of interest to identify new methods to deplete, genetically or through toxins, the entire glial cell population and then study how axons grow across the lesion site.

## Axon regrowth

In addition to neurogenesis and gliogenesis to replace and remodel lost tissue, axons from neurons whose cell bodies are located in the brain project longitudinally across the injury site in the spinal cord. These axons are severed upon injury, but regrow and innervate appropriate targets to restore spinal cord function in adult zebrafish (Becker et al., 1997). Anguita-Salinas et al. recently studied the extent to which regrowing axons follow a pre-injury trajectory. The authors transplanted RFP-tagged cells from a transgenic fish embryo into a *neurod*:*EGFP* embryo that forms EGFP^+^ neurons to generate mosaic animals, and selected larvae with single or few RFP^+^ neurons in the spinal cord. By following the trajectory of RFP-labeled regenerating axons upon injury, they observed different behaviors between ventral and dorsal axons. Specifically, regeneration of dorsal axons was limited or absent, whereas ventral axons regenerated to either ventral or dorsal sites (Anguita-Salinas et al., 2019). The basis of the differential regenerative responses is unclear; however, axonal re-growth is known to be driven in part by intrinsic properties of the neurons themselves, such as pro-regenerative gene expression profiles, as well as by environmental cues that permit axon growth (Rasmussen and Sagasti, 2017).

### Neuron-intrinsic properties

Neurons possess different intrinsic capacities for axonal regrowth after injury (Becker et al., 1997), suggesting that axonal regrowth is, at least in part, driven by cell-autonomous components. Becker et al. identified L1.1, a cell surface protein of the immunoglobulin superfamily, as a candidate cell recognition molecule upregulated in regenerating neurons. Morpholino-based knockdown of L1.1 impaired regrowth of axons from the brainstem as well as locomotor recovery in adult zebrafish (Becker et al., 2004). Phenelzine, an L1 mimetic, increased the expression of L1.1 and phosphorylation of Erk in the caudal region of the lesioned spinal cord, accelerating axon regrowth, remyelination and locomotor recovery (Li et al., 2018). L1.1 is proposed to guide the regenerating axons to their proper targets by homophilic and heterophilic binding with other neuronal cell adhesion molecules such as Axonin-1 and Contactin. Further work including targeted genetic manipulation would refine the specific roles for L1.1 expressed in the brainstem compared to L1.1 expressed in spinal cord interneurons, and would help determine whether L1.1 expression is sufficient to initiate axon growth.

Additional cell recognition molecules have been implicated in axonal regeneration, including Syntenin-a (Yu and Schachner, 2013) and Sema4d (Peng et al., 2017). Interestingly, the latter has been shown to play a role in the interaction between motor neurons and support cells like microglia. Morpholino-mediated knockdown of Sema4D in adult zebrafish decreases microglial association with motor neurons and impairs functional recovery (Peng et al., 2017). Regenerating neurons also upregulate the expression of kinases such as Aurora kinase B, transcription factors such as *atf3* and microRNAs such as miR-133b (Gwee et al., 2018; Wang et al., 2017; Yu et al., 2011b). It is likely that there are many more genes that contribute to cell-autonomous axonal regeneration, and identifying the factors that allow populations of neurons to regrow damaged axons after SCI, for example by expression profiling and molecular genetics, is a priority for the field.

### Environmental properties

After SCI, the regenerating axons must traverse a complex cellular environment to innervate appropriate targets and restore spinal cord function. In mammals, this environment is thought to be non-permissive for axon growth due to the presence of inhibitory ECM components such as chondroitin sulfate proteoglycans (CSPGs) and glial scar tissue formed by reactive astrocytes (Dyck and Karimi-Abdolrezaee, 2015). The cellular environment in the zebrafish, by contrast, is permissive for axon growth.

Macrophages invade the site of SCI and induce an inflammatory response that has been proposed to accelerate axonal recovery in zebrafish larvae (Tsarouchas et al., 2018). An appropriate immune response to injury is expected to be crucial to a pro-regenerative cellular environment. Immune cells such as macrophages and neutrophils are typically responsible for phagocytosing debris such as the degenerating axons and myelin sheaths that remain at the site after SCI. Interestingly, neutrophils are only rarely found near the injury site in adult zebrafish (Goldshmit et al., 2012). In larvae, neutrophils can be observed migrating to the injury site; however, reducing neutrophil migration by genetically ablating microglia has no negative effect on spinal cord regeneration, suggesting that neutrophils are dispensable in spinal cord regeneration (Tsarouchas et al., 2018). Myelin debris can persist in the zebrafish spinal cord to 6 months after injury, and regrowing axons preferentially route through myelin-free gray matter, suggesting alternative, non-phagocytic, roles for macrophages during spinal cord regeneration (Becker and Becker, 2001). Peripheral macrophages can modulate the level of inflammation in the injured spinal cord by increasing Tnf-α production and reducing Il-1β near the injury site. In mutant zebrafish lacking macrophages, regeneration fails due to a prolonged period of inflammation that includes increased exposure to Il-1β (Tsarouchas et al., 2018). Histamine injection in the diencephalon after SCI also increases the level of inflammation, resulting in increased gliosis, i.e. the proliferation or hypertrophy of several different types of glial cells, at the injury site, leading to impaired functional recovery (Huang et al., 2017). Conversely, pharmacological inhibition or genetic ablation of Il-1β in the early stages of regeneration results in its failure, consistent with the notion that inflammation must be tightly regulated for proper regeneration (Tsarouchas et al., 2018).

Axons require not only an appropriate chemical environment for growth, but also a growth-permissive physical substrate. ECM remodeling is thought to be crucial for functional recovery from SCI; for instance, in mice, ECM proteins such as CSPGs are upregulated after injury and can inhibit the growth of axons (Dyck and Karimi-Abdolrezaee, 2015). In zebrafish, morpholino knockdown of Chondroitin-4-sulfotransferase-1, a sulfotransferase important in the synthesis of CSPGs, enhanced the speed of spinal cord regeneration in both larvae and adults, suggesting that this mechanism is conserved between zebrafish and mouse (Sahu et al., 2019). After SCI in zebrafish, the expression of ostensibly growth-permissive ECM components (like Ctgfa) is induced in both neural and non-neural tissues within the injured spinal cord. In larvae, the lesion site itself activates Wnt/β-catenin signaling in fibroblast-like cells, which was reported to increase the deposition of pro-regenerative collagen XII (Wehner et al., 2017). Neurons in the nucleus of median longitudinal fascicle in the brainstem deposit Tenascin-c, an ECM component that directs morphogenetic changes such as neurite growth (Yu et al., 2011a). Morpholino knockdown of Tenascin-c resulted in impaired locomotor recovery and reduced axon growth after SCI. Additionally, *contactin 1a*, which encodes a receptor for Tenascins, is expressed in brainstem neurons after SCI in adult zebrafish, suggesting that, after injury, neurons in the brain are able to activate a genetic program that allows them to remodel the ECM for successful axon growth (Schweitzer et al., 2007) (Fig. 4).

## Angiogenesis

Neurons in the central nervous system have high energy demands, and as such require robust vascularization for proper function (James and Mukouyama, 2011). During development, newly formed neurons and radial glia recruit and coordinate vascular formation in the spinal cord and neighboring tissues through the expression of genes such as *vegfaa* and *flt1* (Matsuoka et al., 2017; Wild et al., 2017). Accordingly, during spinal cord regeneration, the vasculature must re-form properly in concert with CNS tissue. After injury, endothelial cells in the vascular network and motor neurons in the spinal cord each express cell adhesion molecules such as MCAM, which also mediates angiogenesis during tumor formation and development (Chan et al., 2005; Liu et al., 2016). Morpholino knockdown of MCAM during spinal cord regeneration decreases the expression of angiogenesis-related genes and impairs motor recovery, suggesting that MCAM helps to direct angiogenesis after SCI (Liu et al., 2016). Cytokines such as amphoterin (Hmgb1; also known as Hmgb1a) are also expressed in the vasculature after SCI, suggesting that the damaged vascular network is coordinating signaling events in the regenerating spinal cord (Fang et al., 2014). More research is needed to elucidate the mechanisms by which the regenerating spinal cord can recruit and pattern new blood vessels after injury. Genetic tools to target and manipulate factors known to regulate angiogenesis during ontogenetic development may help shed new light on how regenerating tissues are able to organize new blood vessel formation in an adult organism, and identify pro-regenerative interventions.

## Perspectives

Spinal cord regeneration in zebrafish is still understudied and in some ways mysterious, although several advances have been made over the past decade. Cells acting as neuronal progenitors, named ERGs, seem to contribute to regeneration of spinal cord neurons, a process that is limited in mammals. Yet, zebrafish ERGs share several features with mammalian spinal cord stem cells, such as *foxj1* expression (Ribeiro et al., 2017), which is important in specification of certain progenitor subtypes during mammalian CNS development (Jacquet et al., 2009; Jacquet et al., 2011). A deeper understanding of the key gene regulatory networks – the upstream and downstream regulators driving neurogenesis from ERGs in zebrafish – can help uncover species-related differences. After the identification of candidate targets, researchers can perform functional studies using standard genome-editing approaches that have advantages over morpholino-based knockdowns. Additionally, neurogenesis and emergence of movements/behaviors in zebrafish embryos was recently analyzed at a single-cell level using an elegant imaging method for comprehensively tracking neuron lineages (Wan et al., 2019). It is exciting to envision similar imaging strategies to visualize the re-establishment of neuronal pools and restoration of circuits in the regenerating spinal cord.

Several recent studies have described enhancer elements in the genome that are responsive to injury and direct gene expression preferentially in regeneration contexts (Gehrke et al., 2019; Goldman et al., 2017; Harris et al., 2016; Hewitt et al., 2017; Hung et al., 2015; Kang et al., 2016; Suzuki et al., 2019). These sequences, and the regulatory factors that bind to them, have not been probed during spinal cord regeneration. Revealing how pro-regenerative gene expression changes are regulated at the chromatin level can help explain how regeneration is triggered in zebrafish, and it might shed light on the reasons why this does not occur in mammals. Ultimately, deciphering the signals that promote endogenous spinal cord regeneration in zebrafish might help unlock and activate regenerative processes in mammalian contexts.
